# SGLT2 inhibitors and incretin-based therapies for metabolic dysfunction-associated steatohepatitis: a systematic review

**DOI:** 10.1007/s00228-026-04095-7

**Published:** 2026-06-10

**Authors:** Artur Macedo Cruz, Bruna Carolyne Venancio Lima, José Erivelton Souza Maciel de Ferreira, Rian Brito Teles

**Affiliations:** 1 University of Gurupi, Gurupi, Tocantins Brazil; 2 Medical Residency in Internal Medicine, Idomed Quixadá, Ceará, Brazil; 3 Estácio Faculty of Medicine of Juazeiro do Norte, Ceará, Brazil; 4 Federal University of Cariri, Ceará, Brazil; 5 University for the International Integration of the Afro-Brazilian Lusophony (UNILAB), Ceará, Brazil; 6 Abelardo Gadelha da Rocha Municipal Hospital, Caucaia, Ceará, Brazil

**Keywords:** Sodium-Glucose Transporter 2 Inhibitors, Metabolic dysfunction-associated steatohepatitis, Glucagon-Like Peptide-1 Receptor Agonists, Metabolic Diseases, Insulin Resistance

## Abstract

**Background:**

In recent years, metabolic therapies originally developed to treat systemic metabolic disorders have been investigated as potential therapeutic strategies for Metabolic dysfunction-associated steatohepatitis (MASH).

**Objective:**

This study aimed to critically evaluate recent clinical evidence on emerging metabolic therapies, particularly sodium-glucose cotransporter-2 (SGLT2) inhibitors and incretin-based agents, examining their effects on hepatic and metabolic outcomes and, when available, histological endpoints, as well as safety in patients with MASH.

**Methods:**

A systematic review of clinical studies evaluating metabolic therapies in patients with MASH or metabolically associated fatty liver disease was conducted. Randomized clinical trials and other relevant clinical studies investigating SGLT2 inhibitors, incretin-based therapies, and other emerging metabolic agents were included. Outcomes of interest comprised metabolic parameters, hepatic outcomes related to steatosis and disease activity, and histological endpoints when available.

**Results:**

Twelve clinical studies were included. In trials with histological endpoints, semaglutide achieved steatohepatitis resolution without worsening of fibrosis in 62.9% versus 34.3% with placebo in a phase 3 trial (*p* < 0.001), while a phase 2 trial reported NASH resolution in 59% versus 17% with placebo (*p* < 0.001), without significant fibrosis improvement (43% versus 33%; *p* = 0.48). Tirzepatide achieved MASH resolution without worsening of fibrosis in 44–62% of patients versus 10% with placebo (*p* < 0.001 for all doses), and fibrosis improvement in 51–55% versus 30%. Among SGLT2 inhibitors, dapagliflozin achieved MASH improvement without worsening of fibrosis in 53% versus 30% (*p* = 0.006), MASH resolution in 23% versus 8% (*p* = 0.01), and fibrosis improvement in 45% versus 20% (*p* = 0.001). Overall, metabolic therapies improved body weight, hepatic steatosis, glycemic parameters, and disease activity, but evidence for durable fibrosis regression and long-term liver-related outcomes remains heterogeneous.

**Conclusion:**

Emerging metabolic therapies show promising effects on metabolic and hepatic outcomes in patients with MASH. Incretin-based therapies appear to exert particularly robust effects on body weight reduction and steatohepatitis resolution, whereas SGLT2 inhibitors may provide complementary metabolic, cardiometabolic, and hepatic benefits. Nevertheless, fibrosis-related effects remain less consistent across studies, and future trials with paired histological endpoints, longer follow-up, and clinically meaningful liver-related outcomes are needed.

## Introduction

Metabolic dysfunction-associated steatotic liver disease (MASLD), previously known as nonalcoholic fatty liver disease (NAFLD), has become the most prevalent form of chronic liver disease worldwide and is a major cause of liver-related morbidity and mortality. The condition is strongly associated with obesity, insulin resistance, and type 2 diabetes mellitus (T2DM), affecting approximately two thirds of individuals with diabetes and substantially increasing the risk of progression to metabolic dysfunction-associated steatohepatitis (MASH), cirrhosis, hepatocellular carcinoma, and liver-related death [1].

The pathophysiology of MASH is complex and multifactorial, involving systemic metabolic dysregulation, adipose tissue dysfunction, chronic low-grade inflammation, and insulin resistance. These mechanisms contribute to excessive lipid accumulation in the liver, lipotoxicity, oxidative stress, and progression of hepatic fibrosis. Beyond its hepatic complications, MASH is strongly associated with cardiovascular disease, chronic kidney disease, and extrahepatic malignancies, underscoring its systemic metabolic nature [2].

Despite the growing clinical burden of MASH, pharmacological treatment options remain limited. Lifestyle modification, particularly through weight loss and metabolic control, continues to be the cornerstone of therapy. However, sustaining these interventions over the long term is often difficult in routine clinical practice, and only a few pharmacological therapies have been specifically approved for MASH. This scenario has intensified interest in drugs originally developed for metabolic diseases, especially those used to treat diabetes and obesity, that may exert beneficial effects on hepatic metabolism.

Among these agents, sodium-glucose cotransporter-2 (SGLT2) inhibitors have attracted increasing attention because of their metabolic and anti-inflammatory effects and their potential to reduce liver fat. Emerging clinical evidence suggests that these drugs may improve hepatic steatosis, lower liver enzyme levels, and improve metabolic parameters in patients with MASH. Randomized clinical trials and meta-analyses have shown significant reductions in liver fat content assessed by imaging techniques such as magnetic resonance imaging-proton density fat fraction (MRI-PDFF), as well as improvements in indirect markers of hepatic fibrosis after treatment with SGLT2 inhibitors [3, 4]. Likewise, systematic reviews focusing on empagliflozin have reported improvements in insulin resistance, liver stiffness, and aminotransferase levels in patients with NAFLD [5].

Recent clinical studies have also reported promising results with empagliflozin in patients with T2DM and MASH, including significant reductions in liver enzymes, improved imaging scores, and decreases in noninvasive indices of hepatic fibrosis, suggesting a potential therapeutic role for these agents in the management of metabolic liver disease [6]. In addition, observational data from large population-based cohorts indicate that the use of SGLT2 inhibitors may be associated with a greater likelihood of NAFLD regression and a lower incidence of adverse hepatic outcomes compared with other classes of oral antidiabetic agents [7].

In addition to SGLT2 inhibitors, incretin-based therapies, such as glucagon-like peptide-1 (GLP-1) receptor agonists and dual agents such as tirzepatide, have also demonstrated beneficial effects on hepatic steatosis and metabolic parameters. Network meta-analyses comparing different metabolic therapies indicate that these agents may significantly reduce liver fat content and improve liver enzymes, reinforcing the therapeutic potential of metabolism-targeted strategies in MASH [8, 28].

Nevertheless, despite these promising findings, much of the available evidence still relies on metabolic biomarkers, laboratory parameters, and imaging methods, whereas studies directly assessing histological endpoints remain relatively limited. An integrated assessment of hepatic, metabolic, and histological outcomes is essential to understand the true impact of emerging metabolic therapies on MASH progression and clinical outcomes.

Accordingly, this systematic review aimed to critically evaluate recent clinical evidence on emerging metabolic therapies, particularly SGLT2 inhibitors and incretin-based agents, examining their effects on hepatic and metabolic outcomes and, when available, histological endpoints, as well as safety in patients with MASH.

## Methods

### Study design

This systematic review was conducted in accordance with the PRISMA 2020 statement, and the completed PRISMA checklist is provided as supplementary material. The review protocol was prospectively registered in the International Prospective Register of Systematic Reviews (PROSPERO). The review sought to critically synthesize the available evidence on the impact of pharmacological metabolic therapies in the treatment of MASH, with particular emphasis on the effects of sodium-glucose cotransporter-2 inhibitors and incretin-based therapies on histological, metabolic, and clinical outcomes related to the disease.

The research question was structured according to the PICO framework, considering adults with MASH as the population of interest, pharmacological metabolic therapies as the intervention, placebo or standard treatment as the comparator, and histological and metabolic outcomes as the main endpoints. Thus, the review sought to determine whether pharmacological interventions targeting the metabolic axis are capable of promoting resolution of disease activity or improvement in hepatic fibrosis in patients with MASH.

## Search strategy

A comprehensive literature search was conducted in the PubMed/MEDLINE database to identify relevant studies evaluating pharmacological metabolic therapies for metabolic dysfunction-associated steatohepatitis. The search strategy was designed to maximize sensitivity and was developed using a combination of Medical Subject Headings (MeSH) and free-text terms.

The search terms captured the spectrum of metabolic liver disease, including metabolic dysfunction-associated fatty liver disease and metabolic dysfunction-associated steatohepatitis, as well as their previous nomenclature, nonalcoholic fatty liver disease and nonalcoholic steatohepatitis. Corresponding abbreviations (MAFLD, MASH, NAFLD, and NASH) were also incorporated to enhance retrieval.

These terms were combined using Boolean operators with descriptors related to pharmacological interventions targeting metabolic pathways. Specifically, terms for sodium–glucose cotransporter-2 (SGLT2) inhibitors and individual agents (dapagliflozin, empagliflozin, canagliflozin, ertugliflozin, and ipragliflozin) were included. In addition, terms related to incretin-based therapies and emerging metabolic agents were incorporated, including glucagon-like peptide-1 receptor agonists (GLP-1 RAs), semaglutide, liraglutide, tirzepatide, fibroblast growth factor 21 (FGF21), and efruxifermin.

Boolean operators (“AND” and “OR”) were applied to appropriately combine disease- and intervention-related terms. No initial restrictions on study design were applied during the search phase in order to ensure comprehensive identification of relevant studies.

The search was restricted to PubMed/MEDLINE because this database provides extensive coverage of biomedical and pharmacological literature, uses structured indexing through Medical Subject Headings (MeSH), and has strong representation of clinical trials and intervention studies relevant to pharmacological therapy. To reduce retrieval bias within this single-database strategy, the search combined MeSH terms and free-text terms, incorporated both current and previous disease nomenclature, included individual drug names and therapeutic class terms, and applied filters aligned with the review question, including publication period, language, full-text availability, and exclusion of secondary studies. PubMed Clinical Queries and trial-oriented search filters were also considered to improve the identification of intervention studies and clinical trials.

The complete PubMed/MEDLINE search string was as follows: (“Metabolic Dysfunction-Associated Fatty Liver Disease” OR “Metabolic Dysfunction-Associated Steatotic Liver Disease” OR “Metabolic Dysfunction-Associated Steatohepatitis” OR MAFLD OR MASLD OR MASH OR “Nonalcoholic Fatty Liver Disease” OR “Nonalcoholic Steatohepatitis” OR NAFLD OR NASH) AND (“Sodium-Glucose Transporter 2 Inhibitors” OR “SGLT2 inhibitor” OR “SGLT2 inhibitors” OR dapagliflozin OR empagliflozin OR canagliflozin OR ertugliflozin OR ipragliflozin OR “GLP-1 receptor agonist” OR “GLP-1 receptor agonists” OR semaglutide OR liraglutide OR tirzepatide OR “incretin therapy” OR “incretin-based therapy” OR FGF21 OR efruxifermin). The search strategy is presented in Table 1.

**Table 1:** Search strategy used in the PubMed database

Search Strategy Component	Terms Used	Boolean Operator
#1 Metabolic liver disease	“Metabolic Dysfunction-Associated Fatty Liver Disease” OR “Metabolic dysfunction-associated steatohepatitis” OR “Nonalcoholic Fatty Liver Disease” OR “Nonalcoholic Steatohepatitis” OR MAFLD OR MASH OR NAFLD OR NASH	OR
#2 SGLT2 inhibitors	“Sodium-Glucose Transporter 2 Inhibitors” OR “SGLT2 inhibitors” OR dapagliflozin OR empagliflozin OR canagliflozin OR ertugliflozin OR ipragliflozin	OR
#3 Incretin-based therapies	“GLP-1 receptor agonist” OR semaglutide OR liraglutide OR tirzepatide OR “incretin therapy” OR “incretin-based therapy”	OR
#4 Liver-targeted metabolic therapies	FGF21 OR efruxifermin	OR
Final combined strategy	(Metabolic liver disease) AND (SGLT2 inhibitors OR incretin-based therapies OR liver-targeted therapies)	#1 AND (#2 OR #3 OR #4)

Source: Prepared by the authors (2026)

## Eligibility criteria

This review included original studies that investigated the use of pharmacological metabolic therapies in adults diagnosed with MASH or with fatty liver disease associated with clinically relevant metabolic disorders. Eligible studies included randomized clinical trials, interventional studies, and prospective studies published within the past five years that reported clinical, metabolic, or histological data related to the course of liver disease.

Selected studies had to evaluate pharmacological interventions with a potential impact on the systemic metabolic environment and on the pathophysiology of metabolic liver disease, with particular emphasis on sodium-glucose cotransporter-2 inhibitors and incretin-based therapies. Studies reporting clinically relevant disease-related outcomes, such as changes in the histological activity of steatohepatitis, regression or improvement of hepatic fibrosis, reduction of hepatic steatosis, or improvement in metabolic parameters associated with insulin resistance, were also considered.

Review articles, systematic reviews, meta-analyses, clinical guidelines, editorials, letters to the editor, case reports, case series, and experimental or preclinical studies were excluded. Articles that did not present primary data or did not directly address clinical, metabolic, or histological outcomes related to metabolic dysfunction-associated liver disease were also excluded.

## Study selection process

Study selection was conducted independently by two reviewers in two sequential stages using the Rayyan web application. In the first stage, titles and abstracts of all records identified through the literature search were screened according to predefined eligibility criteria. During this phase, blinding between reviewers was enabled to minimize selection bias. Records clearly unrelated to the review question, non-primary studies, preclinical studies, case reports, and studies not reporting relevant hepatic, metabolic, or histological outcomes were excluded. All retrieved records were imported into a reference management software, and duplicate records were identified and removed before screening. Because the search strategy was run using multiple combinations of disease- and intervention-related terms, duplicate records retrieved across search combinations were identified and removed before screening.

In the second stage, the full texts of potentially eligible articles were independently assessed by the same reviewers to confirm eligibility. This assessment considered the characteristics of the study population, the type of pharmacological intervention, the presence of relevant hepatic or metabolic outcomes, and alignment with the review objectives.

**Fig. 1:**
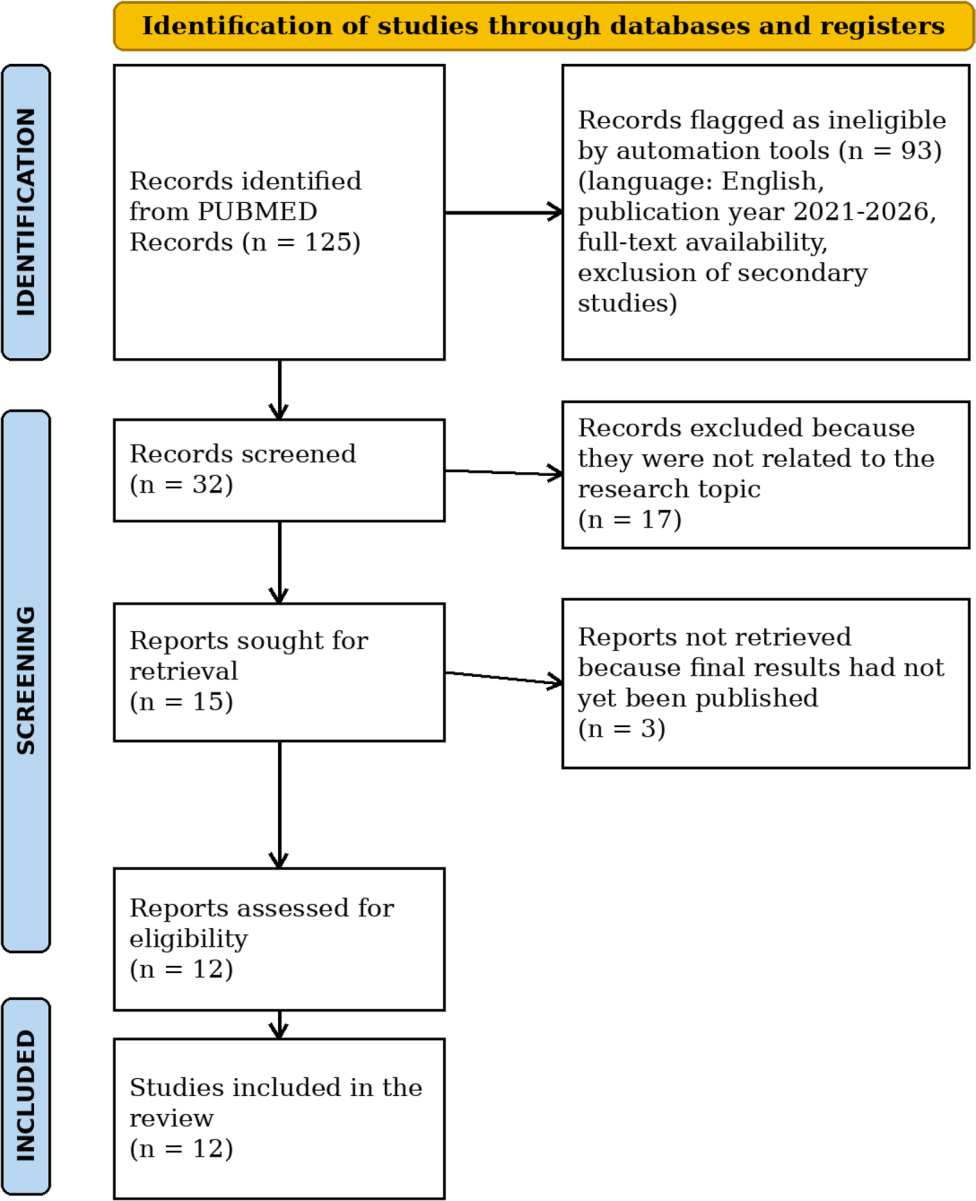
PRISMA flow diagram of the present study. Source: Prepared by the authors (2026)

Disagreements between reviewers were resolved through discussion and consensus. When consensus could not be reached, a third reviewer was consulted. Reasons for exclusion at the full-text stage were documented and are presented in the PRISMA flow diagram (Fig. 1).

## Data extraction

Data extraction from the included studies was performed in a standardized manner to ensure consistency in the comparative analysis of the selected studies. For each study, information was collected on the authors and year of publication, journal, study design, characteristics of the evaluated population, and type of pharmacological intervention used.

Data regarding the comparator, follow-up duration, and main outcomes investigated were also recorded. Particular attention was given to outcomes related to liver disease activity, including resolution of steatohepatitis, improvement in inflammatory activity, or regression of hepatic fibrosis. In addition, metabolic outcomes such as changes in body weight, glycemic control, insulin resistance, and cardiometabolic profile were extracted.

When available, reported adverse events and safety information on the therapies evaluated were also recorded. The extracted data were subsequently organized into a summary table to facilitate comparison across the studies included in the review.

### Methodological quality and risk of bias assessment

The methodological quality of the included studies was considered during the critical interpretation of results, taking into account study design, sample size, presence of a comparator group, methods used to assess hepatic outcomes, and duration of follow-up. Randomized clinical trials were considered to provide higher levels of evidence, particularly when they included histological assessment through liver biopsy or validated methods for evaluating steatosis and fibrosis. Additional methodological aspects, such as intention-to-treat analysis, adequacy of randomization, and clear reporting of eligibility criteria, were also considered.

Methodological quality and potential risk of bias were assessed narratively according to study design. Randomized trials were evaluated using domains aligned with RoB 2, whereas non-randomized, open-label, or observational studies were assessed using domains aligned with ROBINS-I. Given the heterogeneity of designs, populations, interventions, and outcomes, risk of bias was not pooled; instead, methodological limitations were incorporated into the interpretation of the findings, particularly indirectness of evidence, reliance on surrogate endpoints, limited follow-up, and absence of paired histological assessment.

All assessments were performed independently by two reviewers. Disagreements were resolved through discussion and consensus, with involvement of a third reviewer when necessary. The results of the methodological quality and risk of bias assessments were used to inform the interpretation of findings, particularly when comparing histological outcomes with imaging-based, biochemical, or other noninvasive surrogate endpoints in studies involving MASH.

## Synthesis of results

Given the heterogeneity in study designs, populations, and analyzed outcomes, a quantitative meta-analysis was not performed. Therefore, the results were synthesized using a structured narrative approach.

The synthesis sought to identify consistent patterns in the effects of metabolic therapies on hepatic and metabolic outcomes related to metabolic dysfunction-associated liver disease. In addition to the analysis of clinical and histological results, potentially relevant pathophysiological mechanisms underlying the action of the therapies evaluated were considered, as well as their clinical applicability in the management of patients with metabolic liver disease and associated cardiometabolic comorbidities.

## Results

The PubMed/MEDLINE search identified 125 records. Of these, 93 records were flagged as ineligible by automation tools according to predefined filters, including language, publication year, full-text availability, and exclusion of secondary studies. A total of 32 records were screened by title and abstract, and 17 were excluded because they were not related to the research topic or did not meet the predefined eligibility criteria. Fifteen reports were sought for retrieval; however, three reports were not retrieved because final study results had not yet been published. Therefore, 12 full-text reports were assessed for eligibility. All 12 studies met the inclusion criteria and were included in the qualitative synthesis. The complete process of identification, screening, eligibility, and inclusion is shown in Fig. 1. The methodological characteristics of the included studies and their main findings are summarized in Table 2.

**Table 2:** Summary of the included clinical studies evaluating metabolic therapies in metabolic dysfunction-associated liver disease

Author (year)	Therapeutic class	Intervention	Study design, population and risk-of-bias considerations	Main hepatic and metabolic outcomes	Main quantitative findings
Sanyal et al. [9]	Incretin-based therapy	Semaglutide 2.4 mg once weekly	Ongoing phase 3, multicenter, randomized, double-blind, placebo-controlled trial; biopsy-defined MASH with F2–F3 fibrosis. High methodological quality/low risk of bias; main limitation: interim analysis and absence of long-term clinical outcomes.	Steatohepatitis resolution; fibrosis improvement; body weight	At week 72, steatohepatitis resolution without worsening of fibrosis occurred in 62.9% with semaglutide vs. 34.3% with placebo; estimated difference 28.7% points, 95% CI 21.1 to 36.2, *p* < 0.001. Fibrosis reduction without worsening of steatohepatitis occurred in 36.8% vs. 22.4%; estimated difference 14.4% points, 95% CI 7.5 to 21.3, *p* < 0.001. Combined steatohepatitis resolution and fibrosis reduction occurred in 32.7% vs. 16.1%; estimated difference 16.5% points, 95% CI 10.2 to 22.8, *p* < 0.001. Mean body-weight change was − 10.5% vs. − 2.0%; estimated difference − 8.5% points, 95% CI − 9.6 to − 7.4, *p* < 0.001.
Loomba et al. [10]	Dual incretin-based therapy	Tirzepatide 5, 10, or 15 mg once weekly	Phase 2, multicenter, double-blind, randomized, placebo-controlled dose-finding trial; biopsy-confirmed MASH with F2–F3 fibrosis. Moderate to high quality; limited follow-up for long-term fibrosis progression and liver-related outcomes.	MASH resolution; fibrosis improvement	MASH resolution without worsening of fibrosis occurred in 10% with placebo vs. 44%, 56%, and 62% with tirzepatide 5, 10, and 15 mg, respectively. Differences vs. placebo were 34% points; 95% CI 17 to 50, 46% points; 95% CI 29 to 62, and 53% points; 95% CI 37 to 69; *p* < 0.001 for all comparisons. Fibrosis improvement ≥ 1 stage without worsening of MASH occurred in 30% with placebo vs. 55%, 51%, and 51% with tirzepatide 5, 10, and 15 mg, respectively.
Newsome et al. [11]	Incretin-based therapy	Semaglutide 0.1, 0.2, or 0.4 mg once daily	Phase 2, double-blind, randomized, placebo-controlled trial; biopsy-confirmed NASH with F1–F3 fibrosis. Moderate to high quality; fibrosis improvement was not significant and long-term clinical outcomes were not assessed.	NASH resolution; fibrosis improvement; weight loss	NASH resolution without worsening of fibrosis occurred in 40%, 36%, and 59% with semaglutide 0.1, 0.2, and 0.4 mg, respectively, vs. 17% with placebo; *p* < 0.001 for semaglutide 0.4 mg vs. placebo. Fibrosis improvement occurred in 43% with semaglutide 0.4 mg vs. 33% with placebo; *p* = 0.48. Mean weight loss was 13% with semaglutide 0.4 mg vs. 1% with placebo.
Loomba et al. [12]	Incretin-based therapy	Semaglutide 2.4 mg once weekly	Phase 2, double-blind, randomized, placebo-controlled trial; biopsy-confirmed NASH-related compensated cirrhosis. Moderate quality; small sample size and limited power, with no significant benefit for fibrosis or NASH resolution.	Fibrosis improvement; NASH resolution; safety	Fibrosis improvement ≥ 1 stage without worsening of NASH occurred in 11% with semaglutide vs. 29% with placebo; OR 0.28, 95% CI 0.06 to 1.24, *p* = 0.087. There was no significant difference in NASH resolution between groups; *p* = 0.29. Adverse events occurred in 89% vs. 79%, and serious adverse events in 13% vs. 8%. No hepatic decompensation events or deaths were reported.
Alkhouri et al. [13]	Combination metabolic therapy	Semaglutide plus cilofexor and/or firsocostat	Randomized, open-label phase 2 proof-of-concept trial; NASH with F2–F3 fibrosis or MRI-PDFF ≥ 10% and liver stiffness ≥ 7 kPa. Moderate quality with risk-of-bias concerns due to open-label design, exploratory endpoints, and combination therapy.	Safety; MRI-PDFF; liver biochemistry; noninvasive fibrosis tests	A total of 108 patients were randomized. Adverse events occurred in 73–90% across groups, mostly gastrointestinal. Despite similar weight loss of approximately 7–10%, combination regimens produced greater absolute reductions in liver steatosis by MRI-PDFF than semaglutide alone: least-squares mean absolute change − 9.8% to − 11.0% with combination therapy vs. − 8.0% with semaglutide monotherapy. Efficacy outcomes were exploratory.
Noureddin et al. [14]	Liver-directed metabolic therapy/FGF21 analogue	Efruxifermin 28 or 50 mg once weekly	Phase 2b, multicenter, randomized, double-blind, placebo-controlled trial; biopsy-confirmed MASH with F2–F3 fibrosis. Moderate to high quality; phase 3 confirmation is still needed.	Fibrosis improvement; MASH worsening; safety	At week 96, ≥ 1-stage fibrosis improvement without MASH worsening occurred in 19% with placebo, 30% with efruxifermin 28 mg, and 49% with efruxifermin 50 mg in the modified intention-to-treat population. Difference vs. placebo: 12% points, 95% CI − 6 to 31, *p* = 0.19 for 28 mg; and 31% points, 95% CI 12 to 49, *p* = 0.0030 for 50 mg. Among participants with week-96 biopsies, the rates were 24%, 46%, and 75%, respectively.
Lin et al. [15]	SGLT2 inhibitor	Dapagliflozin 10 mg once daily	Multicenter, double-blind, randomized, placebo-controlled trial; biopsy-confirmed MASH. Moderate to high quality; paired biopsy strengthens evidence, although generalizability may be limited by population characteristics.	MASH improvement; MASH resolution; fibrosis improvement	At week 48, MASH improvement without worsening of fibrosis occurred in 53% with dapagliflozin vs. 30% with placebo; RR 1.73, 95% CI 1.16 to 2.58, *p* = 0.006. MASH resolution without fibrosis worsening occurred in 23% vs. 8%; RR 2.91, *p* = 0.01. Fibrosis improvement without MASH worsening occurred in 45% vs. 20%; RR 2.25, *p* = 0.001. Adverse events were similar between groups.
Liu et al. [16]	SGLT2 inhibitor plus lifestyle intervention	Dapagliflozin plus calorie restriction	Multicenter, double-blind, randomized, placebo-controlled trial; overweight/obese patients with type 2 diabetes. High quality for metabolic outcomes, but indirect for MASH because hepatic histology was not assessed.	Diabetes remission; weight; insulin resistance	Diabetes remission occurred in 44% with dapagliflozin plus calorie restriction vs. 28% with calorie restriction plus placebo; RR 1.56, 95% CI 1.17 to 2.09, *p* = 0.002. Body weight reduction was greater with dapagliflozin; between-group difference − 1.3 kg, 95% CI − 1.9 to − 0.7. HOMA-IR also improved more with dapagliflozin; difference − 0.8, 95% CI − 1.1 to − 0.4.
Wang et al. [17]	SGLT2 inhibitor	Dapagliflozin 10 mg once daily	Randomized placebo-controlled clinical trial; patients with type 2 diabetes without known heart failure. Limited for MASH inference due to preprint status, small sample, and systemic inflammatory rather than hepatic outcomes.	Systemic inflammation; cardiac fibrosis markers	Over 12 months, dapagliflozin reduced plasma IL-1β by − 1.8 pg/mL, *p* = 0.003, and increased ketones by 0.26 mM, *p* = 0.0001. PBMC maximal oxygen consumption rate decreased in the placebo group but not with dapagliflozin, supporting a potential anti-inflammatory effect. No significant changes were observed in cardiac extracellular volume or T2 relaxation time.
Borlaug et al. [18]	SGLT2 inhibitor	Dapagliflozin 10 mg once daily	Single-center, double-blind, randomized, placebo-controlled trial; HFpEF. High quality for cardiovascular outcomes, but indirect for MASH because hepatic or MASH-specific endpoints were not assessed.	Hemodynamic and cardiometabolic outcomes	Dapagliflozin reduced pulmonary capillary wedge pressure compared with placebo at rest and exercise; overall PCWP change *p* < 0.001. Estimated treatment difference was − 3.5 mmHg at rest, 95% CI − 6.6 to − 0.4, *p* = 0.029, and − 5.7 mmHg at maximal exercise, 95% CI − 10.8 to − 0.7, *p* = 0.027. Body weight decreased by − 3.5 kg, 95% CI − 5.9 to − 1.1, *p* = 0.006.
Hirayama et al. [19]	SGLT2 inhibitor	Ipragliflozin 50 mg vs. metformin	Prospective, multicenter, open-label, blinded-endpoint randomized controlled study; Japanese patients with type 2 diabetes. Moderate quality; open-label design and reliance on noninvasive steatosis/fibrosis indices limit certainty.	Hepatic steatosis indices; noninvasive fibrosis indices	At 24 weeks, ipragliflozin improved hepatic steatosis indices more than metformin: FLI − 9.24 ± 10.7 vs. − 3.45 ± 11.8, *p* = 0.013; HSI − 1.45 ± 2.32 vs. − 0.45 ± 1.87, *p* = 0.021; NAFLD-LFS − 0.70 ± 1.46 vs. − 0.04 ± 0.98, *p* = 0.011. APRI also improved: −0.110 ± 0.323 vs. 0.033 ± 0.181, *p* = 0.010. Changes in FLI and HSI correlated with visceral fat area reduction.

Source: Prepared by the authors (2026)

In general, the analyzed studies investigated pharmacological interventions with metabolic activity in populations with metabolic liver disease or in individuals at high cardiometabolic risk. The interventions assessed were mainly concentrated in two therapeutic approaches: SGLT2 inhibitor-based therapies and incretin-based therapies. The results are presented according to these two predominant therapeutic strategies.

The narrative assessment of methodological quality and potential risk of bias indicated that randomized controlled trials generally provided the most robust evidence, particularly when they included placebo control, predefined endpoints, and histological assessment. However, some concerns remained regarding follow-up duration, heterogeneity of outcome definitions, reliance on noninvasive or surrogate endpoints, and limited ability to assess long-term fibrosis progression. Non-randomized and open-label clinical studies were interpreted with greater caution because of potential selection bias, lack of blinding, residual confounding, and limitations in attributing observed effects to specific interventions. Therefore, findings from these studies were used mainly as supportive evidence for metabolic and cardiometabolic effects rather than as definitive evidence of histological efficacy in MASH.

### Evidence related to sodium-glucose cotransporter-2 inhibitors

Studies evaluating interventions based on sodium-glucose cotransporter-2 inhibitors consistently showed improvements in metabolic parameters related to metabolic liver disease. The most frequently reported findings included reductions in body weight, improvement in insulin resistance, and overall improvement in cardiometabolic profile, particularly in populations with type 2 diabetes or high cardiometabolic risk [5, 16]. In the study by Liu et al., dapagliflozin plus calorie restriction was associated with a higher rate of diabetes remission than placebo plus calorie restriction, as well as greater reductions in body weight and insulin resistance, supporting its systemic metabolic effects.

In addition to systemic metabolic effects, part of the evidence indicated an impact on hepatic outcomes. The strongest direct evidence for this class came from the dapagliflozin trial in biopsy-confirmed MASH, in which treatment improved MASH activity, MASH resolution, and fibrosis-related histological outcomes compared with placebo. Specifically, dapagliflozin achieved MASH improvement without worsening of fibrosis in 53% of patients versus 30% with placebo, MASH resolution in 23% versus 8%, and fibrosis improvement without worsening of MASH in 45% versus 20% [15]. These findings are clinically relevant because they suggest potential hepatic effects beyond glycemic control, although confirmation in broader and more diverse populations remains necessary.

Similar supportive findings were observed in studies using noninvasive hepatic markers. Ipragliflozin improved hepatic steatosis indices and APRI compared with metformin in patients with type 2 diabetes, suggesting potential beneficial effects on early metabolic liver disease, although the absence of paired histological confirmation limits interpretation [19]. Other studies also suggested systemic anti-inflammatory and cardiometabolic effects of dapagliflozin, including reductions in IL-1β and improvements in hemodynamic parameters in patients with heart failure with preserved ejection fraction [17, 18].

Overall, the included SGLT2 inhibitor studies suggest improvements in metabolic and cardiometabolic parameters, liver fat, inflammatory markers, and, in selected studies, histological activity. However, the strength of evidence varied according to the directness of the population and outcomes assessed. Studies with biopsy-confirmed MASH provide more direct evidence of hepatic efficacy, whereas studies focused on diabetes remission, systemic inflammation, cardiovascular outcomes, or noninvasive liver indices should be interpreted as supportive rather than definitive evidence of histological efficacy in MASH.

### Evidence related to incretin-based therapies

The included studies also evaluated the impact of incretin-based therapies in the treatment of metabolic dysfunction-associated liver disease, including GLP-1 receptor agonists and dual agonists with broader metabolic action. Overall, these interventions demonstrated significant metabolic effects, particularly regarding weight loss, improvement in insulin resistance, and better glycemic control [9, 10].

Randomized clinical trials with histological endpoints showed higher rates of steatohepatitis resolution compared with placebo. Semaglutide demonstrated a consistent effect on steatohepatitis resolution in both phase 2 and phase 3 trials. In the phase 3 trial, steatohepatitis resolution without worsening of fibrosis occurred in 62.9% of patients receiving semaglutide versus 34.3% receiving placebo, while fibrosis reduction without worsening of steatohepatitis occurred in 36.8% versus 22.4% [9]. In the earlier phase 2 trial, NASH resolution without worsening of fibrosis occurred in 59% of patients receiving semaglutide 0.4 mg versus 17% with placebo, although fibrosis improvement was not statistically significant [11].

Tirzepatide also showed robust effects in patients with biopsy-confirmed MASH and moderate or severe fibrosis, with MASH resolution without worsening of fibrosis occurring in 44–62% of treated patients versus 10% with placebo, and fibrosis improvement in 51–55% versus 30% [10]. These results suggest that dual incretin agonism may provide clinically meaningful effects on both inflammatory disease activity and fibrosis-related endpoints, although larger and longer trials are still needed to confirm durability and long-term clinical benefit.

However, incretin-based therapies did not show uniform effects across all disease stages. In patients with NASH-related compensated cirrhosis, semaglutide did not significantly improve fibrosis or NASH resolution compared with placebo, with fibrosis improvement occurring in 11% of patients receiving semaglutide versus 29% receiving placebo [12]. This finding reinforces that therapeutic response may differ according to baseline fibrosis stage, disease severity, and duration of follow-up.

In addition to histological outcomes, incretin-based and combination metabolic therapies improved hepatic steatosis and biochemical parameters. Combination therapy with semaglutide, cilofexor, and/or firsocostat produced additional reductions in MRI-PDFF compared with semaglutide alone, although the open-label proof-of-concept design and exploratory endpoints limit causal interpretation [13]. Efruxifermin, a liver-directed FGF21 analogue, also showed improvement in fibrosis-related histological outcomes at longer follow-up, particularly at the 50 mg dose, with fibrosis improvement of at least one stage without MASH worsening in 49% of patients versus 19% with placebo at week 96 [14].

The included studies therefore support improvements in metabolic parameters, hepatic steatosis, and steatohepatitis activity in metabolic dysfunction-associated liver disease, with more consistent results for inflammatory disease activity and weight reduction than for durable fibrosis regression. The quantitative estimates summarized in Table 2 further show that the magnitude and certainty of effect vary across therapeutic classes, study designs, populations, and outcome definitions.

## Discussion

The analyzed studies indicate that pharmacological interventions aimed at modulating systemic metabolism may exert a clinically relevant impact on several dimensions of the disease, including reduction of hepatic steatosis, improvement in metabolic parameters, and, in some trials, improvement in histological endpoints.

The biological plausibility of these findings is consistent with current knowledge regarding the pathophysiology of MASH. The disease is characterized by a set of interrelated metabolic abnormalities, including insulin resistance, adipose tissue dysfunction, low-grade systemic inflammation, and hepatocellular lipid overload, all of which contribute to progression from steatosis to inflammation and hepatic fibrosis [2]. In this context, therapies capable of modulating the systemic metabolic milieu may act on central determinants of metabolic liver disease progression.

Among these approaches, SGLT2 inhibitors have received growing attention because of their ability to induce broad metabolic changes, including modest weight loss, improved insulin resistance, and reduction of systemic metabolic inflammation. Mechanistic evidence suggests that these agents may reduce hepatic lipogenesis, stimulate fatty acid oxidation, and attenuate intrahepatic inflammatory and oxidative processes, while also influencing mechanisms related to gut microbiota and cellular autophagy [20, 21]. These mechanisms provide a plausible pathophysiological basis for the effects observed in clinical studies involving patients with metabolic liver disease.

Across the clinical evidence evaluated, SGLT2 inhibitors demonstrated consistent benefits in both metabolic and hepatic parameters. In a randomized clinical trial involving patients with biopsy-confirmed MASH, dapagliflozin was associated with a higher proportion of improvement in disease activity without worsening of fibrosis, as well as a higher rate of steatohepatitis resolution compared with placebo [22]. Complementarily, clinical studies in populations with type 2 diabetes showed reductions in hepatic steatosis and improvements in noninvasive fibrosis markers after the use of SGLT2 inhibitors [19]. Although the number of studies with direct histological assessment remains limited, these findings suggest that the metabolic modulation promoted by this class may also affect hepatic inflammatory activity.

In addition to hepatic effects, the analyzed studies indicate relevant systemic metabolic benefits associated with SGLT2 inhibitors, including improved glycemic control, weight loss, and reductions in inflammatory markers ([16]; WANG et al., 2024). These effects are especially relevant considering the systemic nature of metabolic dysfunction-associated liver disease, in which cardiometabolic abnormalities play a central role in disease progression and in the development of extrahepatic complications.

Evidence from large-scale observational studies also suggests that the use of SGLT2 inhibitors may be associated with reductions in clinically important outcomes in patients with metabolic liver disease. Population-based cohort studies have shown lower risks of mortality, hepatic decompensation, and cardiovascular events among users of this drug class, reinforcing the potential clinical impact of these interventions beyond traditional metabolic outcomes [23–25].

More recently, however, pharmacological development for MASH has been strongly driven by incretin-based therapies and other emerging metabolic agents. Clinical trials involving GLP-1 receptor agonists have shown significant improvements in inflammatory disease activity and higher rates of steatohepatitis resolution compared with placebo [11]. Nevertheless, early results suggested more modest effects on fibrosis regression, indicating that resolution of inflammatory activity may precede more durable structural changes in hepatic tissue [12].

More recent studies involving dual agonists, such as tirzepatide, have shown more robust results for both MASH resolution and improvement in hepatic fibrosis, possibly reflecting the greater magnitude of weight loss and systemic metabolic improvement associated with these agents [9, 10]. Likewise, combination therapies targeting different metabolic pathways have also improved hepatic and metabolic parameters in patients with metabolic liver disease [13].

Weight loss appears to be a central mediator of the histological and metabolic benefits observed with these therapies. Recent comparative evidence suggests that improvement in MASH activity and fibrosis is strongly influenced by the magnitude of weight reduction, particularly among incretin-based and multi-incretin therapies. This finding is clinically relevant because it indicates that the hepatic benefit of metabolic therapies may not depend exclusively on direct intrahepatic pharmacological effects, but also on sustained improvement in the systemic metabolic environment [26].

Beyond these strategies, agents directed at specific hepatic metabolic pathways have shown promising results in recent clinical studies. The FGF21 analog efruxifermin demonstrated significant improvement in disease activity and histological fibrosis improvement in a phase 2b trial, suggesting that interventions targeting intrahepatic metabolic mechanisms may exert a more direct impact on fibrogenic processes [14].

Recent meta-analyses also support the therapeutic potential of these approaches. GLP-1-based therapies have shown significant reductions in liver fat and improvements in inflammatory histological parameters, although their impact on fibrosis remains more variable across studies [17]. Similarly, pooled analyses of clinical trials involving SGLT2 inhibitors have demonstrated significant improvement in hepatic steatosis and in noninvasive fibrosis markers, although with moderate magnitude and heterogeneity across the available studies [4, 15].

A clear distinction should be made between improvement in steatosis, reduction in inflammatory activity, histologically confirmed regression of hepatic fibrosis, and long-term liver-related clinical benefit. Although changes in liver fat content, aminotransferase levels, MRI-PDFF, elastography, and noninvasive fibrosis scores are clinically relevant and useful for assessing hepatic steatosis, fibrosis risk, and disease activity, they should not be interpreted as equivalent to paired histological improvement or durable clinical benefit. Reductions in imaging-based or biochemical parameters may not necessarily translate into lower risks of cirrhosis progression, hepatic decompensation, hepatocellular carcinoma, liver transplantation, or liver-related mortality, particularly in the absence of paired biopsy confirmation and long-term follow-up. In the current evidence base, fibrosis outcomes remain more heterogeneous and less consistent than outcomes related to weight loss, glycemic control, hepatic steatosis, and steatohepatitis activity [27]. This limitation is particularly relevant when interpreting studies of sodium-glucose cotransporter-2 inhibitors, which frequently rely on surrogate endpoints rather than paired liver biopsy [27].

Consistent with this, SGLT2 inhibitors and incretin-based therapies should not be considered equivalent within the category of “metabolic therapies.” Although both target systemic metabolic dysfunction, the available evidence differs in scope and consistency. Incretin-based therapies show more consistent effects on body weight reduction and steatohepatitis activity, particularly in randomized trials with histological endpoints. In contrast, SGLT2 inhibitors show stronger evidence for metabolic, glycemic, renal, and cardiometabolic outcomes, while hepatic effects are more often based on imaging, liver enzymes, or noninvasive markers. These differences suggest that these therapies may have complementary roles rather than interchangeable effects in the management of MASH.

### Conceptual model of therapeutic axes in MASH

The synthesis of the available evidence supports a conceptual model in which emerging therapies for MASH can be organized into three main therapeutic axes, defined according to the predominant level at which they intervene in disease pathophysiology.

The first axis involves interventions aimed at systemic metabolic control, acting primarily on metabolic disturbances broadly associated with the disease, such as insulin resistance, hyperglycemia, and dysregulated lipid metabolism. The second axis comprises therapies capable of modulating energy balance and inducing significant weight loss, a factor recognized as a key determinant in reducing hepatic inflammatory activity. The third axis includes pharmacological approaches directed at liver-specific metabolic pathways, acting more directly on intrahepatic inflammatory, metabolic, and fibrogenic processes.

This conceptual framework suggests that emerging therapies for MASH should not be interpreted only in isolation, but rather as interventions that operate at different levels of a complex systemic disease. In this context, future disease management may involve combination therapeutic strategies capable of acting simultaneously across these different pathophysiological levels and potentially producing more robust effects on disease activity, fibrosis progression, and associated cardiometabolic risk.

Despite the growing therapeutic potential of pharmacological metabolic agents, sustained lifestyle intervention remains the cornerstone of management in patients with MASH. Weight loss achieved through dietary modification, physical activity, and control of cardiometabolic risk factors continues to play a central role in reducing hepatic steatosis and inflammatory activity. Pharmacological therapy should therefore be considered complementary to, rather than a replacement for, structured lifestyle interventions. In patients unable to maintain long-term lifestyle changes, prolonged pharmacological treatment may be required to sustain metabolic and hepatic benefits, raising important considerations regarding treatment duration, adherence, cost, and long-term safety.

### Limitations of the available evidence

Several limitations should be considered when interpreting the evidence currently available. The main histological trial involving dapagliflozin was conducted predominantly in an Asian population, which may limit the generalizability of its findings to other populations. In addition, the relatively short duration of some studies limits the evaluation of long-term clinical outcomes, such as progression to decompensated cirrhosis, hepatocellular carcinoma, or liver-related mortality.

Another important issue is the heterogeneity of the endpoints assessed across the available studies. Many trials rely on surrogate markers, imaging methods, or noninvasive biomarkers to assess steatosis and fibrosis, whereas only a limited number of studies include histological assessment confirmed by liver biopsy. This methodological heterogeneity makes direct comparison across different pharmacological interventions more difficult.

Another methodological limitation is that the literature search was restricted to PubMed/MEDLINE. Although PubMed provides broad coverage of biomedical and pharmacological literature, the exclusion of Embase, CENTRAL/Cochrane Library, Scopus, and Web of Science may have reduced the sensitivity of study capture, particularly for conference abstracts, European pharmacological literature, and trials indexed outside MEDLINE. Therefore, publication bias and database-selection bias cannot be fully excluded.

### Future perspectives

Taken together, the available data suggest that future MASH treatment will likely move toward a more individualized and mechanism-based approach. Patient phenotype, presence of type 2 diabetes, degree of obesity, cardiovascular and renal risk, baseline fibrosis stage, and likelihood of sustained weight loss may influence therapeutic selection. In this context, incretin-based therapies may be particularly relevant for patients in whom obesity and inflammatory disease activity predominate, whereas SGLT2 inhibitors may be especially attractive in individuals with type 2 diabetes, cardiovascular disease, heart failure, or chronic kidney disease risk.

Combination strategies may also become increasingly relevant, as MASH results from overlapping metabolic, inflammatory, and fibrogenic pathways. Future trials should clarify whether combining systemic metabolic therapies, weight-loss-promoting agents, and liver-directed therapies can produce additive or synergistic effects on steatohepatitis resolution, fibrosis regression, and long-term clinical outcomes. Longer follow-up, paired histological assessment, standardized noninvasive biomarkers, and clinically meaningful endpoints will be essential to define the definitive position of these pharmacological classes in the management of MASH.

## Conclusion

Emerging metabolic therapies show promising effects on metabolic and hepatic outcomes in patients with MASH. Incretin-based therapies appear to exert particularly robust effects on body weight reduction and disease activity, while SGLT2 inhibitors may provide complementary metabolic and cardiometabolic benefits. However, fibrosis-related effects remain less consistent across studies, highlighting an important gap in the current literature. Overall, these findings reinforce the relevance of targeting the metabolic axis in MASH, but also suggest that no single therapeutic class is likely to fully address the complexity of the disease. Future clinical trials with paired histological endpoints, longer follow-up, and clinically meaningful liver-related outcomes are needed to better define the magnitude and durability of treatment effects and to establish the optimal role of these therapies in MASH management.

## Data Availability

No datasets were generated or analysed during the current study.
